# A Sterile Folliculocentric Acneiform Eruption Mimicking Infection After Hair Transplantation: Clinical Pattern Recognition and a Proposed FUE Epidermal Cap–Angle Mechanism

**DOI:** 10.1111/jocd.71209

**Published:** 2026-09-20

**Authors:** Jae Hyun Park

**Affiliations:** ^1^ Dana Plastic Surgery Clinic Seoul Republic of Korea

**Keywords:** acneiform eruption, antimicrobial stewardship, follicular unit excision, hair transplantation, postoperative complications, sterile folliculitis

## Abstract

**Background:**

Acneiform eruption following hair transplantation is frequently encountered in clinical practice but remains incompletely characterized. Because these pustular eruptions may resemble postoperative infection, they often lead to unnecessary antibiotic use.

**Aims:**

To characterize the clinical pattern of a sterile folliculocentric acneiform eruption after hair transplantation and to propose a hypothesis‐based mechanistic model integrating graft morphology and implantation geometry.

**Patients/Methods:**

In this retrospective observational cohort study, 76 consecutive patients who developed acneiform eruption were analyzed. Clinical features, eruption timing, anatomic distribution, graft composition, and microbiologic findings were evaluated.

**Results:**

Mean onset occurred at postoperative day (POD) 3.1 (SD 1.1), with 84% of cases presenting between POD 2–4. Eruptions were predominantly observed in multi‐hair graft areas following FUE procedures and were strictly confined to the transplanted recipient zone. Among 35 affected patients who also received lateral acute‐angle single‐hair grafts, those lateral zones were spared in 91.4% (32/35), an intra‐individual comparison supporting an angle‐dependent pattern. Wound cultures (*n* = 8) showed no pathogenic bacterial growth. Lesions were confined to the transplanted recipient area and resolved with conservative management.

**Conclusions:**

This sterile folliculocentric eruption appears to represent a technique‐associated inflammatory reaction that may mimic infection in the early postoperative period. To our knowledge, this is the first characterization of this discrete, early‐onset sterile entity as distinct from previously described post‐transplant folliculitis, supporting a non‐antibiotic, stewardship‐oriented management approach. The proposed epidermal cap–angle mechanism is presented as a testable hypothesis.

## 1 Introduction

Hair transplantation is widely performed in dermatologic practice and is generally considered safe [1]. In the early postoperative period, transient inflammatory reactions may occur in the recipient area. Among these, a pustular eruption confined to the transplanted region is frequently observed but incompletely described in the dermatologic literature.

These eruptions are often presumed to represent bacterial folliculitis or surgical‐site infection. As a result, patients may receive systemic antibiotics despite the absence of systemic signs of infection. However, clinical observation suggests that a subset of these reactions may represent a sterile folliculocentric inflammatory phenomenon rather than a true infectious process.

A clearer characterization of this eruption is clinically important, both to improve diagnostic accuracy and to avoid unnecessary antimicrobial therapy. The present study describes the clinical features of this postoperative acneiform eruption and proposes a mechanistic model based on graft morphology and implantation geometry.

## 2 Materials and Methods

### 2.1 Study Design

This study was designed as a retrospective observational cohort study of consecutive patients who developed acneiform eruption following hair transplantation during the defined study period. The analysis focused on clinical pattern recognition and hypothesis‐driven mechanistic interpretation rather than incidence or prevalence estimation.

During the study period, a total of 1272 hair transplantation procedures were performed at our institution. The present analysis includes only patients in whom acneiform eruption was clinically documented through follow‐up evaluation or photographic confirmation.

### 2.2 Patient Selection and Definition of Acneiform Eruption

Patients were included if they developed folliculocentric erythematous papules or pustules confined to the transplanted recipient area within the early postoperative period.

For the purposes of this study, acneiform eruption was defined as sterile, folliculocentric erythematous papules or pustules localized to the recipient area, typically occurring between postoperative Days 2 and 4, without clinical or microbiologic evidence of bacterial infection.

Patients were excluded if they had:

- Evidence of active scalp infection prior to surgery.
- Clinically apparent folliculitis involving the recipient area before transplantation.
- Eruptions occurring outside the transplanted region.
- Immediate postoperative diagnosis of bacterial infection requiring systemic antibiotic therapy

### 2.3 Surgical Technique

Hair transplantation procedures were performed using either follicular unit excision (FUE), follicular unit transplantation (FUT), or combined techniques.

In FUE procedures, grafts were implanted without trimming of the epidermal portion, resulting in retention of an epidermal cap at the graft surface. In FUT procedures, grafts were routinely trimmed into a teardrop‐shaped configuration prior to implantation, thereby reducing retained epidermal tissue. For the purposes of this study, the epidermal cap was defined as the cylindrical epidermal collar retained at the surface of an untrimmed FUE graft, corresponding to the punch diameter. Implantation angle was categorized as relatively vertical or acute based on the recipient‐site incision design and graft orientation rather than by direct instrumental measurement, and therefore represents an approximate, technique‐based estimate. FUE harvesting used punches of 0.9 mm in approximately 90% of cases and 0.8–0.85 mm in the remainder.

Implantation angle was determined intraoperatively according to standard hairline design principles. Frontal and mid‐scalp regions were typically implanted at relatively more vertical angles, whereas temporal and lateral hairline regions were implanted at acute or oblique angles to achieve a natural hair direction. Implantation angle was inferred from operative records and postoperative photographic documentation rather than quantitatively measured.

### 2.4 Data Collection

The following variables were collected from operative records and follow‐up documentation:

- Patient sex
- Surgical method (FUE, FUT, or combined)
- Graft composition (single‐hair vs. multi‐hair grafts)
- Postoperative day of eruption onset
- Anatomical distribution of lesions.
- Baseline scalp characteristics, including oily scalp and a history of clinically diagnosed seborrheic dermatitis (recorded separately from, and not synonymous with, simple oily scalp)
- Treatment modality
- Clinical course and time to improvement

Baseline sebaceous tendency was assessed based on patient‐reported history and clinical evaluation at the time of consultation. No objective quantitative sebum measurement was performed.

### 2.5 Microbiologic Evaluation

Wound cultures, including aerobic and anaerobic bacterial cultures (and fungal cultures when clinically indicated), were performed in the initial eight patients presenting with pronounced pustular lesions. Following consistent results demonstrating no bacterial growth and the recognition of a highly reproducible clinical pattern that resolved rapidly with simple mechanical decompression, routine bacterial cultures were deemed unnecessary for subsequent typical cases. Cultures were thereafter reserved for atypical or unusually severe presentations because eight consecutive cultures from the most severe cases were uniformly negative, and the clinical course was stereotyped and self‐limited; universal culture sampling was not clinically indicated.

### 2.6 Treatment Approach

Management consisted primarily of mechanical decompression using rolling pressure with a sterile cotton swab directed along the axis of hair growth, followed by topical application of povidone–iodine. This intervention was applied based on clinical judgment.

In three patients with particularly severe erythema and swelling, a short course of oral minocycline was additionally given as a precautionary measure; it was not used routinely once mechanical decompression was recognized as reliably effective.

The described interventions represent hypothesis‐driven, experience‐based management approaches rather than established standards of care.

### 2.7 Outcome Assessment

Clinical improvement and graft retention were assessed clinically and photographically at routine postoperative follow‐up visits through the early hair‐growth period. No apparent compromise in graft retention was observed in the treated areas.

### 2.8 Implantation Angle Assessment

The implantation angle (vertical versus acute) for each graft was evaluated visually during the postoperative clinical examinations. For the purpose of this clinical evaluation, an acute implantation angle was defined as ≤ 30 degrees relative to the skin surface, whereas a vertical angle was defined as > 30 degrees. In hair transplantation, implantation angles are largely standardized according to the anatomical recipient region to ensure natural hair flow. For instance, strictly acute angles are routinely utilized in the lateral and temporal hairlines, whereas more vertical angles are often required in specific dense‐packing areas. Because these regional techniques dictate the angle, the distinction between an acute and vertical insertion is readily discernible upon direct visual inspection of the recipient site, allowing for accurate retrospective clinical correlation with the eruption patterns.

### 2.9 Ethical Considerations

This retrospective study was granted exemption from full ethical review by an institutional review board; the requirement for individual informed consent for the retrospective analysis of de‐identified records was waived on this basis, and the full approval details (approving committee and reference number) are provided on the title page in accordance with the journal's double‐blind policy. In addition, written informed consent for the publication of clinical photographs and video was obtained from the patients.

## 3 Results

### 3.1 Patient Characteristics and Surgical Context

A total of 76 consecutive patients who developed acneiform eruption following hair transplantation were included. During the same study period, 1272 hair transplantation procedures were performed at our institution (Table 1). This study was not designed to determine incidence or prevalence. Patient demographics and surgical characteristics are summarized in Table 1.

**TABLE 1:** Patient characteristics and surgical context (*n* = 76).

Variable	Value
Sex	Male 59 (77.6%), Female 17 (22.4%)
Surgical method	FUE‐based 67 (88.2%), FUT 7 (9.2%), Combined 2 (2.6%)
Total institutional volume during study period	1272 cases

*Note:* Demographic characteristics and surgical methods of the 76 patients with acneiform eruption after hair transplantation.

### 3.2 Timing of Eruption

Acneiform eruption occurred at a relatively consistent postoperative time point. The mean onset was postoperative day 3.05 ± 1.06, with most eruptions occurring between postoperative Days 2 and 4 (Table 2).

**TABLE 2:** Temporal characteristics of acneiform eruption.

Variable	Value
Mean onset (POD)	3.05 ± 1.06
Median onset (POD)	3
Range (POD)	2–7
Peak onset window	Postoperative Days 2–4

*Note:* Timing of acneiform eruption onset after hair transplantation, expressed as postoperative day (POD).

### 3.3 Anatomic Distribution and Graft Type

In all patients, acneiform eruption was confined to the recipient area and presented primarily as folliculocentric erythematous papules or pustules.

Lesions occurred predominantly in areas transplanted with multi‐hair grafts. In contrast, only 7 patients (9.2%) developed lesions in frontal hairline areas transplanted exclusively with single‐hair grafts.

### 3.4 Lateral Areas With Single‐Hair Grafts

Within the cohort, 35 affected patients had concurrently received single‐hair grafts implanted at an acute angle in lateral recipient areas such as the temple, infratemple, and sideburn during the same operative session. Although all 35 developed eruption in the centrally located, vertically implanted multi‐hair zones, the lateral acute‐angle single‐hair areas were involved in only 3 patients (8.6%) and spared in 32 patients (91.4%). In all three involved cases, the severity was minimal, limited to 1–3 small lesions (Table 3). Because these comparisons were made within the same patients undergoing a single operative session, they provide an intra‐individual control for host‐level and perioperative factors.

**TABLE 3:** Distribution of acneiform eruption in lateral recipient areas.

Variable	Value
Affected patients who also received lateral acute‐angle single‐hair grafts	35
Lateral acute‐angle zones involved	3 (8.6%)
Lateral acute‐angle zones spared	32 (91.4%)
Severity of eruption	1–3 mild lesions

*Note:* Occurrence of acneiform eruption in lateral recipient areas implanted with single‐hair grafts at acute angles.

### 3.5 Baseline Scalp and Skin Characteristics

Baseline analysis revealed that 30 patients (39.5%) reported an oily scalp, and 15 patients (19.7%) had a history of clinical seborrheic dermatitis (Table 4).

**TABLE 4:** Baseline scalp and skin characteristics.

Characteristic	*n* (%)
Oily scalp	30 (39.5%)
History of clinical seborrheic dermatitis	15 (19.7%)

*Note:* Baseline scalp and skin characteristics of patients who developed acneiform eruption. Seborrheic scalp defined as clinically increased sebaceous activity without inflammatory scaling.

### 3.6 Treatment and Clinical Course

Wound cultures were performed on the initial eight patients, consistently demonstrating no bacterial growth. No empiric antibiotic therapy was initiated prior to culture sampling in these cases. Because subsequent patients presented with a highly reproducible clinical pattern that resolved rapidly with targeted mechanical decompression, routine bacterial cultures were deemed unnecessary. Following a single session of rolling mechanical decompression with a sterile cotton swab and topical povidone–iodine, lesions resolved within 1 day in approximately 95% of patients; a small minority required one additional decompression on the following day (Figure 1; Video 1). In three patients with particularly severe erythema and pustulation, a short course of oral minocycline was additionally given as a precautionary measure; once rapid resolution with mechanical decompression was recognized, systemic antibiotics were no longer used routinely. Clinical stabilization was achieved in all cases, and no apparent compromise in graft survival was observed on routine follow‐up.

**FIGURE 1:**
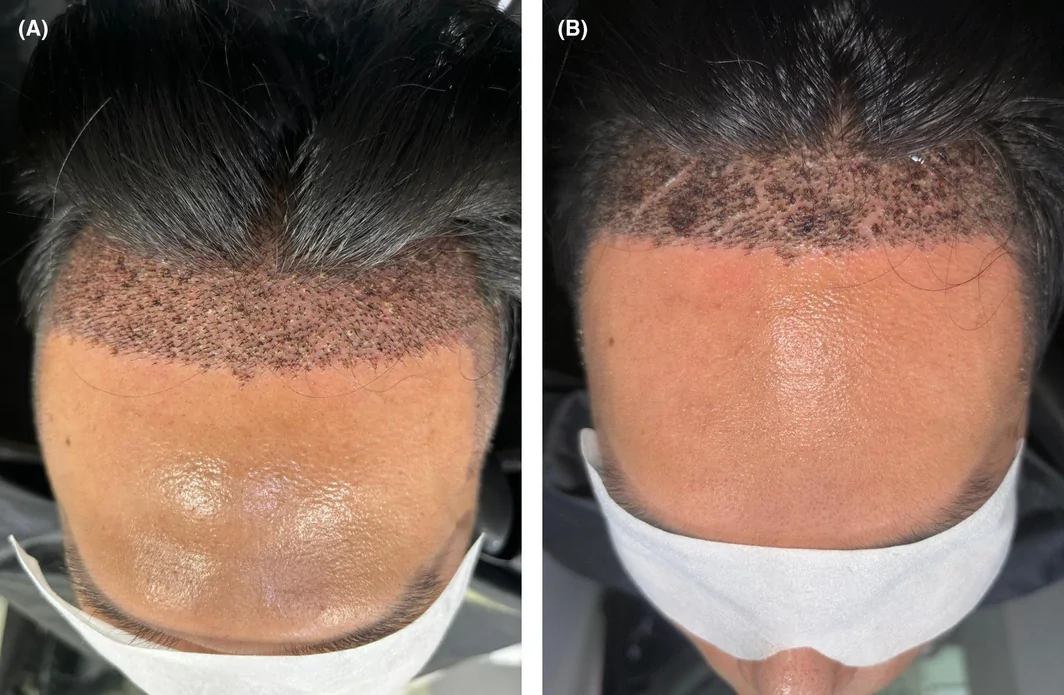
Sterile folliculocentric acneiform eruption after hair transplantation. Clinical presentation and resolution. (A) Postoperative Day 3 showing folliculocentric erythematous papules and pustules confined to the transplanted recipient area. (B) Postoperative Day 4 demonstrating clinical improvement following rolling mechanical decompression and topical povidone–iodine application.

**VIDEO 1:** Rolling mechanical decompression technique. Demonstration of rolling mechanical decompression using a sterile cotton swab directed along the axis of hair growth, followed by topical antiseptic application. Video content can be viewed at https://onlinelibrary.wiley.com/doi/10.1111/jocd.71209.

## 4 Discussion

It is important to note that this is a descriptive case series focused on clinical pattern recognition rather than an epidemiologic study of incidence or prevalence. The central finding of this study is the identification of a technique‐associated, non‐infectious acneiform eruption following hair transplantation. We hypothesize that this phenomenon is primarily related to the “epidermal cap” retained in untrimmed FUE grafts. Unlike FUT grafts, where the manual slivering process typically trims the upper epidermis into a teardrop shape, FUE grafts often retain a cylindrical epidermal collar corresponding to the punch diameter. This structural characteristic may act as a physical “seal,” partially or completely obstructing the follicular ostium and impeding the natural outflow of sebum (Figure 2).

**FIGURE 2:**
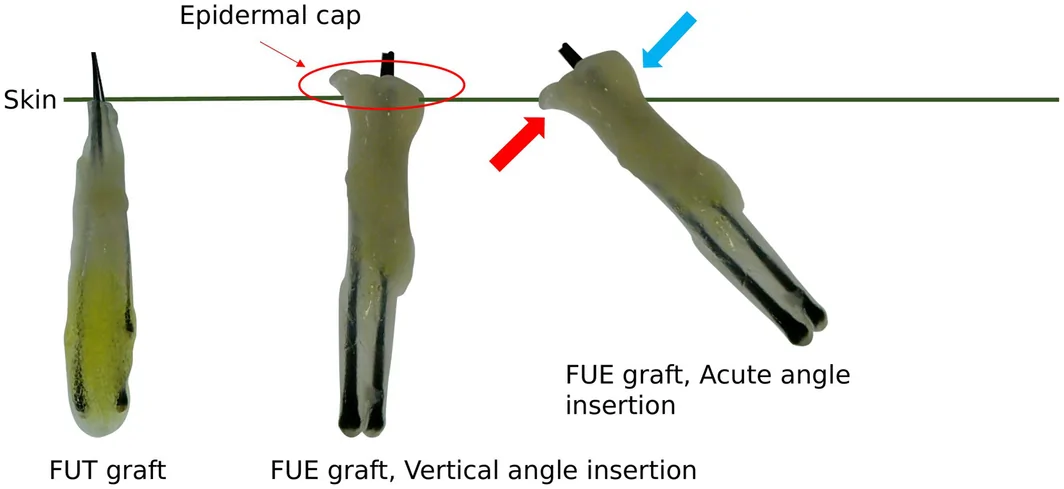
Sterile folliculocentric acneiform eruption after hair transplantation. Proposed mechanistic model based on graft morphology and implantation angle. Schematic illustration demonstrating morphologic differences between follicular unit transplantation (FUT) and follicular unit excision (FUE) grafts and the potential effect of implantation angle. FUE grafts retain an epidermal cap at the surface, whereas FUT grafts are typically trimmed prior to implantation. With relatively vertical insertion, the retained epidermal cap may more directly overlie the follicular ostium (red arrow). In contrast, acute‐angle insertion may allow partial preservation of ostial exposure (blue arrow). This schematic represents a conceptual model illustrating the proposed mechanism and is not a histologic section. The schematic directly illustrates the central hypothesis of this study: that a retained epidermal cap, when combined with a relatively vertical implantation angle, may occlude the follicular ostium and impede sebum outflow, whereas an acute angle preserves partial ostial patency and regional sparing.

This temporal clustering may coincide with early postoperative changes in sebaceous and inflammatory activity. While we previously considered this timeline to coincide with the rapid recovery of sebaceous gland activity, an equally plausible and perhaps concurrent mechanism is an exaggerated acute wound‐healing response [2]. During this early postoperative window, the intense infiltration of neutrophils surrounding the newly implanted follicles in response to localized surgical trauma may synergistically manifest as sterile pustules. The mechanical obstruction by the epidermal cap might effectively exacerbate this normal inflammatory cascade by preventing the natural drainage of exudate and sebum. Baseline sebaceous characteristics, such as an oily scalp or a clinical history of seborrheic dermatitis, likely function as permissive factors facilitating this clinical expression [3, 4].

Our clinical observations suggest an angle‐associated modulation of this obstructive mechanism. In cases where grafts were implanted at vertical angles (> 30°), the epidermal cap more directly covered the follicular opening, maximizing the likelihood of entrapment (Figure 2). Conversely, in the same patients, the lateral areas such as the temples that received single‐hair grafts at acute angles (≤ 30°) were involved in only 8.6% (3/35) and spared in 91.4%. Because this contrast arises within individual patients operated on in a single session, it functions as an intra‐individual control that holds host‐level factors—such as oily scalp, seborrheic tendency, and wound‐healing response—essentially constant, thereby strengthening the inference that graft composition and insertion geometry, rather than patient predisposition alone, drive the regional distribution. From a mechanical perspective, an acute implantation angle prevents the epidermal cap from perfectly sealing the ostium; instead, it creates a ‘lateral gap’ or a skewed opening that allows for partial drainage even under the pressure of the cap [5]. This surgical and anatomical distinction explains why the frontal hairline and temporal regions remain relatively spared compared to more vertically implanted dense‐packing areas (Figure 3). Furthermore, the higher prevalence in multi‐hair grafts compared to single‐hair grafts underscores the impact of the total sebaceous burden.

**FIGURE 3:**
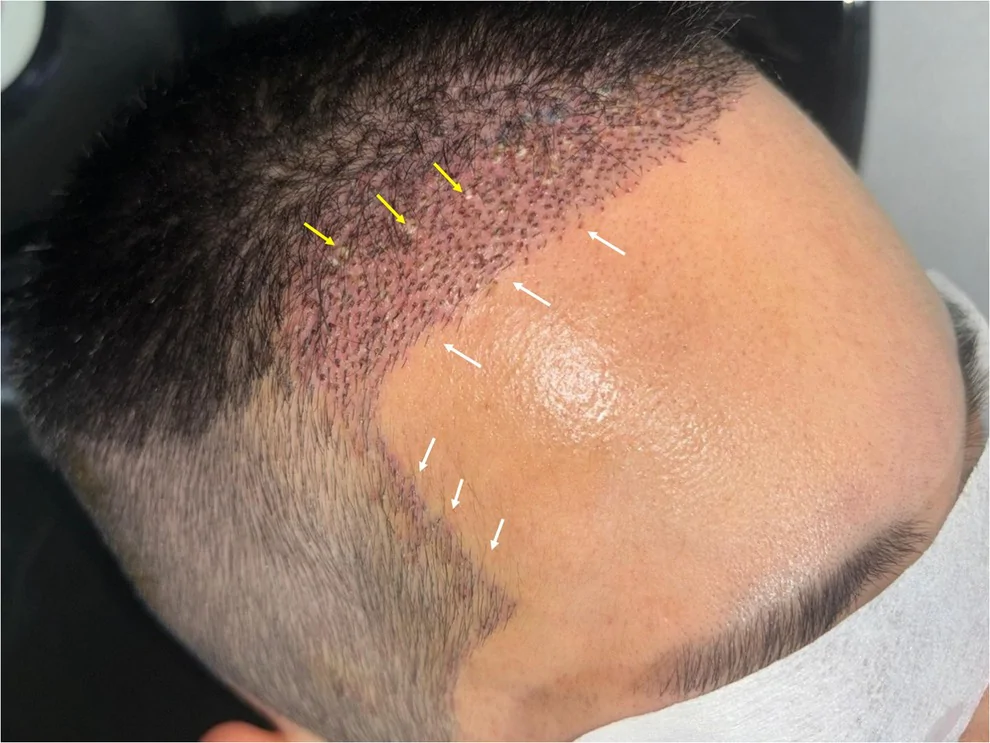
Sterile folliculocentric acneiform eruption after hair transplantation. Distribution pattern in relation to graft type and implantation angle. Distribution pattern of acneiform eruption on postoperative day 3. Yellow arrows indicate pustular lesions predominantly observed in areas transplanted with multi–follicular unit grafts. White arrows indicate relative sparing in regions transplanted with single‐hair grafts in the frontal and temporal hairline. Folliculocentric pustules with surrounding erythema are visible within the affected zone.

Our findings differ distinctly from previously described postoperative folliculitis. For instance, Bunagan et al. [6] reported recipient area folliculitis following FUT, which typically presented with a delayed onset ranging from several days to months postoperatively. Recent multicenter and scoping studies [7, 8, 9] have broadly characterized postoperative folliculitis and sterile folliculitis as common but heterogeneously defined complications after hair transplantation, emphasizing macro‐level surgical and postoperative care factors without delineating specific morphologic subtypes or implantation‐angle–dependent mechanisms. In contrast, the present series defines an early‐onset (84% between POD 2–4), strictly folliculocentric sterile eruption confined to dense, vertically implanted FUE recipient zones and proposes a mechanistic model integrating retained epidermal caps, graft composition, and insertion geometry, thereby offering a technique‐targetable explanation and a simple, non‐antibiotic management strategy.

Alternative or contributing mechanisms should also be considered. Inadvertent implantation of graft‐associated debris—epidermal skin tags, crushed epidermis or fat, or fragments of transected hair shafts adherent to the graft—can provoke a sterile foreign‐body inflammatory reaction as the retained material is extruded, and such debris‐related inflammation may contribute to, or coexist with, the proposed epidermal cap mechanism. Meticulous graft preparation to minimize retained debris may therefore reduce the occurrence of these eruptions.

Historically, prior to defining this specific sterile acneiform eruption, various empirical treatments were trialed for similar postoperative presentations, including broad‐spectrum systemic antibiotics, yielding minimal clinical benefit. Recognizing this condition as a mechanically exacerbated, non‐infectious process is critical. Our consistent findings of no bacterial growth in wound cultures, combined with the rapid resolution following simple mechanical decompression, are consistent with a mechanically obstructive process. From a practical standpoint, this non‐infectious eruption should be suspected when sterile pustules appear acutely, predominantly in FUE zones with multi‐hair grafts implanted vertically. In our experience, simple mechanical decompression using a sterile cotton swab combined with a topical antiseptic was effective (Figure 1, Video 1); we offer this as a pragmatic, experience‐based approach rather than an established standard of care, pending prospective evaluation. Systemic antibiotics are generally unnecessary for this sterile process and, if considered at all, should be reserved for atypical or unusually severe presentations.

Preventive considerations may include technical modifications such as careful epidermal trimming when feasible and the use of relatively acute implantation angles where cosmetically appropriate. In addition, a non‐touch foaming approach during postoperative Days 1–4 may theoretically assist in softening retained epidermal cap tissue and facilitating sebum clearance, although this remains experience‐based rather than evidence‐validated.

From a clinical perspective, early recognition of the characteristic temporal window (postoperative Days 2–4) and distribution pattern (predominantly multi‐hair graft regions with relative sparing of acute‐angle single‐hair areas) may assist in distinguishing this sterile reaction from bacterial infection. Conservative management, including rolling mechanical decompression and topical antisepsis, appeared sufficient in most cases. In selected patients with more pronounced inflammatory features, oral minocycline was administered primarily for its anti‐inflammatory properties rather than presumed antimicrobial effect. Technical considerations such as graft trimming and implantation angle optimization may theoretically reduce the likelihood of ostial obstruction, although this remains speculative.

From a health‐economic and stewardship perspective, recognizing this eruption as a benign, self‐limited process allows substitution of low‐cost mechanical decompression and topical antisepsis for empiric systemic antibiotic courses. Beyond direct drug costs, avoiding unnecessary antibiotics reduces the risks of adverse drug reactions (including minocycline‐associated vestibular symptoms and hyperpigmentation), gut dysbiosis, and selection for antimicrobial resistance.

In summary, this work advances the field in three principal ways. First, it delineates a discrete, early‐onset (postoperative Days 2–4) sterile folliculocentric eruption that is distinct from the previously described, often later‐onset and heterogeneous post‐transplant folliculitis, including the multicenter series reported by Zhou et al. and the complication reviews of Nadimi and Liu et al. Second, it proposes an intra‐individual, surgical‐geometry framework—retained follicular unit excision (FUE) epidermal caps and implantation angle—to explain regional lesion sparing, offering a testable anatomic hypothesis rather than attributing pustules solely to host seborrhea or postoperative care. Third, it provides a non‐antibiotic, first‐line management approach that directly supports antimicrobial stewardship in a setting where empiric antibiotics are frequently prescribed for misrecognized surgical‐site infection. These contributions are presented as hypothesis‐generating and warrant prospective, controlled validation.

## 5 Limitations

This study was a retrospective single‐center analysis. Although the regional sparing of acute‐angle single‐hair zones was observed within individual patients—providing a degree of intra‐individual control for graft type and implantation angle—no parallel cohort of patients without eruption was systematically analyzed. The associations with oily scalp and seborrheic dermatitis, in particular, should therefore be interpreted as hypothesis‐generating rather than causal. Furthermore, objective morphometric assessment of epidermal cap dimensions and precise instrumental measurement of insertion angles were not performed, which precludes strict quantitative correlation. Finally, baseline sebaceous tendency was assessed based on clinical history rather than objective quantitative measurements. Therefore, our mechanistic explanations should be interpreted as hypothesis‐generating. In addition, other factors that may contribute to the observed regional distribution—such as recipient‐site packing density, graft handling, and local recipient‐site characteristics—were not systematically controlled and cannot be excluded. As a single‐center study using a standardized in‐house technique, the findings may not generalize to centers using different punch sizes, epidermal‐trimming protocols, or postoperative washing regimens, and prospective multi‐center validation is warranted. Punch diameters were narrow and strongly clustered (0.9 mm in approximately 90% of cases; 0.8–0.85 mm in the remainder), and epidermal cap dimensions were not recorded per lesion; the limited variation in punch size precluded a meaningful stratified analysis by cap or punch size. Treatment outcomes were assessed clinically and photographically rather than by standardized quantitative endpoints (such as time‐to‐resolution or pustule counts), and comparative outcome data between management approaches were not systematically recorded. The absence of a matched non‐eruption control cohort further limits epidemiologic and risk inference, and formal case–control quantification of independent risk factors remains a goal for future work.

## 6 Conclusion

Post–hair transplantation acneiform eruption appears to represent a reproducible clinical pattern consistent with a technique‐associated inflammatory reaction. We hypothesize that it results from the complex interplay of retained epidermal caps in untrimmed FUE grafts, implantation angle geometry, baseline sebaceous tendency, and early postoperative acute wound‐healing responses. Reconceiving this entity as a mechanically exacerbated sterile inflammation rather than an infectious complication provides a rational, evidence‐based foundation for its postoperative management.

## Author Contributions

J.H.P. is the sole author and contributed to study conception and design, data collection, data analysis, interpretation of results, drafting and critical revision of the manuscript, and final approval of the version to be published.

## Funding

The author has nothing to report.

## Ethics Statement

The author confirm that the ethical policies of the journal, as noted on the journal's author guidelines page, have been adhered to. This study was conducted in accordance with the principles of the Declaration of Helsinki. The study protocol was reviewed by the Public Institutional Review Board Designated by the Ministry of Health and Welfare (Republic of Korea), which granted exemption from full ethical review (IRB No. P01‐202607‐01‐054).

## Consent

Written informed consent was obtained from the patient(s) for publication of their photographs, video, and clinical information in print and online, with the understanding that this information may be publicly available.

## Conflicts of Interest

The author holds patents related to follicular unit excision punch technology and hair graft implanter devices (Rainbow implanter/graMAX; Seson Medical). These interests had no role in the design, conduct, data analysis, or interpretation of this study, and the author declares no other conflicts of interest.

## Data Availability

The data supporting the findings of this study are available from the corresponding author upon reasonable request.
